# Research progress on the role and dysregulation of NK cells in anti-tuberculosis immunity

**DOI:** 10.3389/fimmu.2026.1917564

**Published:** 2026-09-03

**Authors:** Yaqi Zheng, Fuxiang Li, Bo Zhang, Xiaoling Wang, Zhigang Jiao, Xuebiao Wu, Tong Yang, Jiaxing Deng, Yina Xiong, Huang Zhiqin, Junyun Huang

**Affiliations:** 1 Department of Laboratory Medicine, First Affiliated Hospital of Gannan Medical University, Ganzhou, Jiangxi, China; 2 College of Medical Technology, Gannan Medical University, Ganzhou, Jiangxi, China; 3 The First Clinical Medical College, Gannan Medical University, Ganzhou, Jiangxi, China; 4 Gannan Medical University, Ganzhou, Jiangxi, China

**Keywords:** clinical research, innate immunity, macrophages, NK cells, tuberculosis

## Abstract

Tuberculosis (TB) is one of the most common infectious diseases caused by Mycobacterium tuberculosis (M.tb). The emergence of multidrug-resistant strains, HIV infection, and poor efficacy of the BCG vaccine have made TB epidemics difficult to control. Natural killer (NK) cells are an important component of the body’s innate immunity, playing a role not only in the early stages of M.tb infection but also in activating adaptive immune cells to inhibit M.tb proliferation. Recent studies have increasingly revealed significant changes in the number, subsets, and functions of NK cells as TB progresses, with NK cells primarily exhibiting signs of depletion, suggesting a close correlation between NK cell changes and TB progression. Therefore, this article outlines the main changes in NK cells in clinical cohort studies, discusses potential mechanisms of their depletion, and introduces the functions of NK cells, as well as the methods and strategies they use to recognize and kill Mycobacterium tuberculosis. This can contribute to a better understanding of the role and manifestation of NK cells in TB immunity and provide a more comprehensive perspective for NK cell-based TB treatment strategies.

Tuberculosis (TB), caused by Mycobacterium tuberculosis (M.tb), remains a a major global public health challenge. In 2024, approximately 10.7 million people developed TB worldwide, resulting in approximately 1.23 million deaths (1). Most individuals infected with M. tuberculosis remain in a latent tuberculosis infection (LTBI) state, and approximately 5–10% of individuals with LTBI will eventually develop active TB (2). Host immunity plays a critical role in determining the outcome of M.tb infection. NK cells are key effector cells of innate immunity and are widely distributed in peripheral blood and lymphatic organs, while also residing in tissues such as the liver and lungs, where they contribute to tissue-specific innate immune responses (3). Unlike cytotoxic T lymphocytes (CTLs), NK cells recognize and eliminate target cells without MHC restriction and can mount rapid responses without prior antigen sensitization, making them important mediators of early host defense before the development of adaptive immunity. NK cells can directly contribute to the elimination of intracellular M. tuberculosis through the release of cytotoxic molecules, including granzymes (GZMs), and can also lyse or modulate M. tuberculosis-infected cells through multiple mechanisms. Furthermore, they can interact with and modulate dendritic cells (DCs) and T cells, thereby promoting the development of adaptive immune responses following infection. Collectively, accumulating evidence supports an important role for NK cells in host defense against TB. However, despite their important role in anti-tuberculosis immunity, accumulating evidence indicates that NK cells undergo substantial alterations during TB progression, including changes in their frequency, subset distribution, and functional capacity following M. tuberculosis infection (4). Nevertheless, the mechanisms underlying these alterations in NK-cell number, subset distribution, and function remain incompletely elucidated. This review summarizes current clinical evidence on changes in NK-cell frequency, subset distribution, and function; discusses the potential mechanisms underlying NK-cell dysregulation; and examines the roles and responses of NK cells during M. tuberculosis infection.

## 1 Characterization of NK cells in clinical tuberculosis patients

### 1.1 Changes in the number of NK cells in tuberculosis patients

Given the crucial role of NK cells in in host defense against *Mycobacterium tuberculosis (M.tb)* infection, their phenotypic and functional characteristics have been extensively investigated in clinical studies. In general, TB progression is associated with alterations in NK-cell frequency, subset distribution, and functional capacity, which may contribute to impaired antimycobacterial responses and an exhausted or dysfunctional NK-cell phenotype. A meta-analysis of multiple TB patient cohorts showed that, following *M. tuberculosis* infection, peripheral blood NK-cell numbers increase during the latent phase, decline before or during progression to active TB, and return toward levels observed in healthy individuals after treatment. However, the reported changes in T- and B-cell numbers vary across study populations. Therefore, the authors proposed that changes in NK-cell numbers may serve as a potential indicator of progression from latent infection to active TB (4). Subsequent studies have also reported that peripheral blood NK-cell numbers are lower in patients with active TB than in individuals with LTBI (5) or healthy controls (6, 7). However, other studies have found no significant difference in peripheral blood NK-cell numbers between patients with active TB and uninfected individuals (8).

In addition to overall NK-cell frequency, the distribution of specific NK-cell subsets is also altered during TB progression. The CD3^-^CD7^+^GZMB^+^ NK-cell subset gradually decreases in frequency from healthy controls (HC) to individuals with LTBI and further to patients with active TB, whereas its frequency increases following anti-tuberculosis treatment (9). Compared with healthy individuals, the frequencies of the CD56+CD16+/- subsets (10) and CD8α+NK subsets (11) in peripheral blood were significantly reduced in patients with TB, whereas those of the CD226^+^ NK (12) and HLA-DR+NK (13) subsets were increased. Compared with individuals with LTBI, the frequency of the KLRG1^+^ NK-cell subset in peripheral blood was significantly reduced in patients with active TB (14), while the CD56^neg^ NK-cell subset expressing early- or intermediate-to-late maturation markers was also decreased (5).

### 1.2 NK cell function is affected in tuberculosis patients

Clinical studies have demonstrated that *M. tuberculosis* infection also impairs NK-cell function. Compared with healthy individuals, peripheral blood NK cells from patients with active TB exhibit reduced expression of activating receptors, including NKp30 (10) and NKp46 (9). Single-cell RNA-seq profiling revealed marked upregulation of multiple inhibitory immune checkpoint genes, including *PDCD1* (PD-1), *CTLA4, HAVCR2*, and *BTLA*, in NK cells from patients with active TB. Compared with patients with mild TB, NK cells from patients with severe TB showed increased expression of genes associated with T-cell exhaustion, including *PTPN6, PTPN11*, and *PRDM1*. Concurrently, these cells exhibited increased expression of chemotaxis-associated genes (*CXCL13, CXCL10, CCL3*, and *CCL25*) and pro-apoptotic genes (*FAS, TNF*, and *IRF1*), collectively indicating pronounced NK-cell dysregulation (14). Another single-cell sequencing study reported reduced expression of genes associated with T-cell and B-cell activation in NK cells from patients with TB (15). IFN-γ is an important mediator of host resistance to *M. tuberculosis* infection; however, several studies have reported reduced IFN-γ production by peripheral blood NK cells in patients with pulmonary TB (5, 6, 16), with one study showing that this capacity was partially restored following anti-tuberculosis treatment (17).

Compared with healthy controls (HC) and individuals with LTBI, both the frequencies and absolute numbers of GZMA^+^, GZMB^+^, and GZMK^+^ NK cells were significantly reduced in patients with active TB. Among patients receiving anti-tuberculosis treatment, both measures increased significantly following successful treatment (18).

Meanwhile, multiple studies have shown that NK cells from patients with TB exhibit reduced cytotoxic activity against *M. tuberculosis*-infected monocytes (19, 20), which may be associated with reduced expression of the adhesion molecule CD11a (LFA-1) on NK cells. Reduced CD11a expression may impair immune synapse formation between NK cells and infected target cells, thereby disrupting lytic granule polarization and diminishing NK-cell degranulation and cytotoxicity (16, 20). *In vitro*, IFN-α stimulation of NK cells from patients with active TB improved NK-cell function and partially restored the expression of activating receptors such as NKp30; however, receptor expression remained lower than that observed in healthy controls under the same conditions, indicating persistent NK-cell dysfunction during active TB (10, 19). Other studies have shown that NK-cell numbers and function are further reduced in patients with multidrug-resistant TB compared with those with drug-susceptible TB, whereas NK-cell cytotoxicity can be restored following treatment (21).

### 1.3 NK Cells in tuberculous pleural effusion

Studies characterizing NK cells in tuberculous pleural effusion remain limited. Schierloh et al. found that the total number of NK cells was decreased in tuberculous pleural effusion (PF), whereas the proportion of the CD56^bright^CD16^neg^ subset was relatively increased. They suggested that heat-labile soluble factors present in tuberculous PF selectively downregulated anti-apoptotic genes, such as Bcl-2, in the CD56^dim^CD16^pos^ subset, thereby promoting apoptosis of this cytotoxic subset. Such factors were not detected in pleural effusions from patients with other types of pneumonia (19). Other studies have also found that BCG can lead to apoptosis of NKp44+CD56bright NK cells (22). The reduced number of NK cells in PF may also be associated with their migration toward lymphoid tissues. CD62L and CCR7 are receptors involved in lymph node homing, and their expression is increased on NK cells in the PF of patients with TB (19). In another study, CD56^dim^ NK cells in the peripheral blood of patients with TB also expressed CCR7 (10), further suggesting that *M. tuberculosis* infection may promote NK-cell trafficking from the peripheral blood and PF toward lymphoid tissues. In addition, the expression of the inhibitory receptors CD96 and NKG2A was increased on NK cells in the PF of patients with TB, potentially contributing to reduced NK-cell activity (19).

### 1.4 Potential mechanisms of NK cell depletion in Mtb infection

Although multiple clinical cohort studies have shown that Mtb infection leads to an exhaustion phenotype of NK cells, the underlying mechanisms are less studied. Recent studies have identified leukocyte immunoglobulin-like receptor B1 (LILRB1) as an inhibitory immune checkpoint receptor that drives NK-cell exhaustion during tuberculosis. Mtb-infected macrophages highly express human leukocyte antigen-G (HLA-G), which upregulates and activates LILRB1 on NK cells. HLA-G binding to LILRB1 triggers SHP-1/2-mediated inhibitory signaling, resulting in suppression of MAPK signaling and impaired NK-cell function. Disrupting the interaction between HLA-G and LILRB1 by using anti-LILRB1 blocking antibodies can restore NK cell-dependent anti-tuberculosis immunity in immunized humanized mice (7). Furthermore, the immunosuppressive microenvironment in tuberculosis, characterized by elevated levels of TGF-β and IL-10, as well as increased IDO activity, may further suppress NK-cell activation and effector functions, thereby contributing to NK-cell dysfunction. Current evidence suggests that several mechanisms may contribute to the altered number and impaired function of NK cells during tuberculosis, including: (i) impaired NK-cell development and maturation, resulting in altered subset distribution; (ii) enhanced inhibitory receptor signaling and/or reduced activating receptor signaling; (iii) metabolic dysregulation, including impaired glycolysis; and (iv) regulation by other immunosuppressive cells, such as regulatory T cells (Tregs) and myeloid-derived suppressor cells (MDSCs).

Multiple clinical studies have consistently demonstrated that patients with active tuberculosis exhibit reduced circulating NK-cell numbers, altered subset distribution, and impaired effector function, although the underlying mechanisms remain incompletely understood (4, 6, 7, 18). Given the crucial role of NK cells in anti-tuberculosis immunity, and considering factors such as the emergence of multidrug-resistant strains, HIV infection, and the poor prophylactic efficacy of the BCG vaccine, NK cells that can kill target cells without sensitization have become extremely attractive targets for host immunity. Further research is needed to explore the mechanisms by which Mtb infection suppresses NK cell function, providing another powerful means to end the tuberculosis epidemic as soon as possible (**Table 1**).

**Table 1:** Biological mechanisms underlying NK-cell dysfunction during tuberculosis and their potential host-directed therapeutic strategies.

Biological mechanism	Representative molecules/signaling pathways	Major consequences for NK cells	Potential host-directed therapeutic strategies	References
Immune checkpoint-mediated inhibition	PD-1/PD-L1, Tim-3, TIGIT, CD96, LILRB1, NKG2A/HLA-E axis	NK-cell exhaustion, reduced IFN-γ production, impaired degranulation and cytotoxicity, diminished antimycobacterial activity	Immune checkpoint blockade (particularly targeting LILRB1, NKG2A, and PD-1/PD-L1), combined with cytokine stimulation	(7, 23)
Immunosuppressive cytokine microenvironment	IL-10, TGF-β, impaired IL-12/IL-15/IL-18 signaling, type I interferon signaling	Suppressed NK-cell activation, reduced proliferation, impaired cytokine production and cytotoxic function	Cytokine supplementation (IL-12, IL-15, IL-18, IFN-α); modulation of immunosuppressive cytokine networks	(24, 25)
Altered NK-cell differentiation and maturation	Impaired transition from CD56^bright^ to CD56^dim^ NK cells, expansion of CD56^neg^ subsets, KLRG1-, CD57-, and NKG2C-associated maturation pathways	Imbalanced NK-cell subset composition, accumulation of functionally immature NK cells, reduced cytotoxic capacity.	Cytokine-based strategies to restore NK-cell maturation (not yet clinically validated in tuberculosis)	(5, 25)
Altered tissue trafficking and compartmental redistribution	CXCR3, CCR5, CX3CR1, CD11a/LFA-1, chemokine gradients within pulmonary lesions	Relative depletion of circulating NK cells, preferential recruitment to infected tissues, altered immune surveillance	Modulation of chemokine signaling pathways (currently at the experimental stage)	(19, 24)
Crosstalk with immunosuppressive immune cells	Regulatory T cells (Tregs), myeloid-derived suppressor cells (MDSCs)	Impaired NK-cell activation, proliferation and cytotoxicity, maintenance of an immunosuppressive microenvironment	Targeting suppressive myeloid cells or immunosuppressive cytokine networks	(26)

## 2 Functions and functional regulation of NK cells

Peripheral blood lymphocytes contain approximately 10-15% NK cells. They are derived from hematopoietic stem cells. During differentiation and maturation, NK cells gradually lose the expression of lineage markers, including CD3, CD14, and CD19, while acquiring NK-cell-associated receptors. Mature NK cells are generally defined as CD3^neg^CD56^pos^ lymphocytes. Based on the level of CD56 expression, they are further classified into CD56^bright^ and CD56^dim^ subsets, representing relatively immature cytokine-producing NK cells and more mature highly cytotoxic NK cells, respectively (27) (**Table 2**).

**Table 2:** Phenotypic characteristics and functional features of major NK-cell subsets in tuberculosis.

NK-cell subset	Phenotypic markers	Major functional characteristics	Relevance to TB	Representative reference
CD56^bright NK cells	CD3^-^CD56^bright, usually CD16^−/low	Strong cytokine-producing capacity, particularly IFN-γ; relatively limited cytotoxicity at steady state	CD56^bright NK cells show altered frequency and phenotype in TB; M. tuberculosis-specific CD56^bright responses have also been reported.	(28)
CD56^dim NK cells	CD3^-^CD56^dimCD16^+	Predominantly cytotoxic; efficient degranulation and perforin/granzyme-mediated target-cell killing	Represents the major circulating cytotoxic NK-cell population; its frequency and receptor phenotype can be altered during TB	(28)
CD56^neg NK cells	CD3^-^CD56^negCD16^+ with NK-associated receptors	Functionally heterogeneous; frequently associated with altered cytotoxicity and receptor expression in chronic infection.	Increased/expanded CD56^neg NK cells have been reported particularly in TB/HIV co-infection and are associated with functional impairment.	(29)

Among NK-cell activating receptors, NKG2D, NKp46, NKp44, NKp30, and CD16 have been the most extensively investigated in tuberculosis and are known to play essential roles in the recognition of Mtb-infected cells and the activation of NK-cell effector functions (30–32). NK-cell activation depends on the integration of signals delivered by activating and inhibitory receptors. Among the inhibitory receptors implicated in tuberculosis, KIRs and CD94-NKG2A have been the most extensively investigated (31). Upon engagement of activating receptors, protein tyrosine kinases (PTKs) initiate several canonical signaling pathways. Vav1-dependent actin remodeling primarily regulates NK-cell polarization, whereas the PI3K–PLCγ pathway promotes granule exocytosis. In parallel, MAPK signaling, particularly ERK activation, contributes to cytokine production. Conversely, inhibitory receptors recruit protein tyrosine phosphatases (PTPs) to suppress these activation signals (33–35). These signals ultimately drive the expression and release of NK cell effector molecules, among which granzyme B (GZMB) is one of the core effector molecules mediating NK cell cytotoxicity and a crucial molecule determining its anti-infection and killing functions.

**Figure 1:**
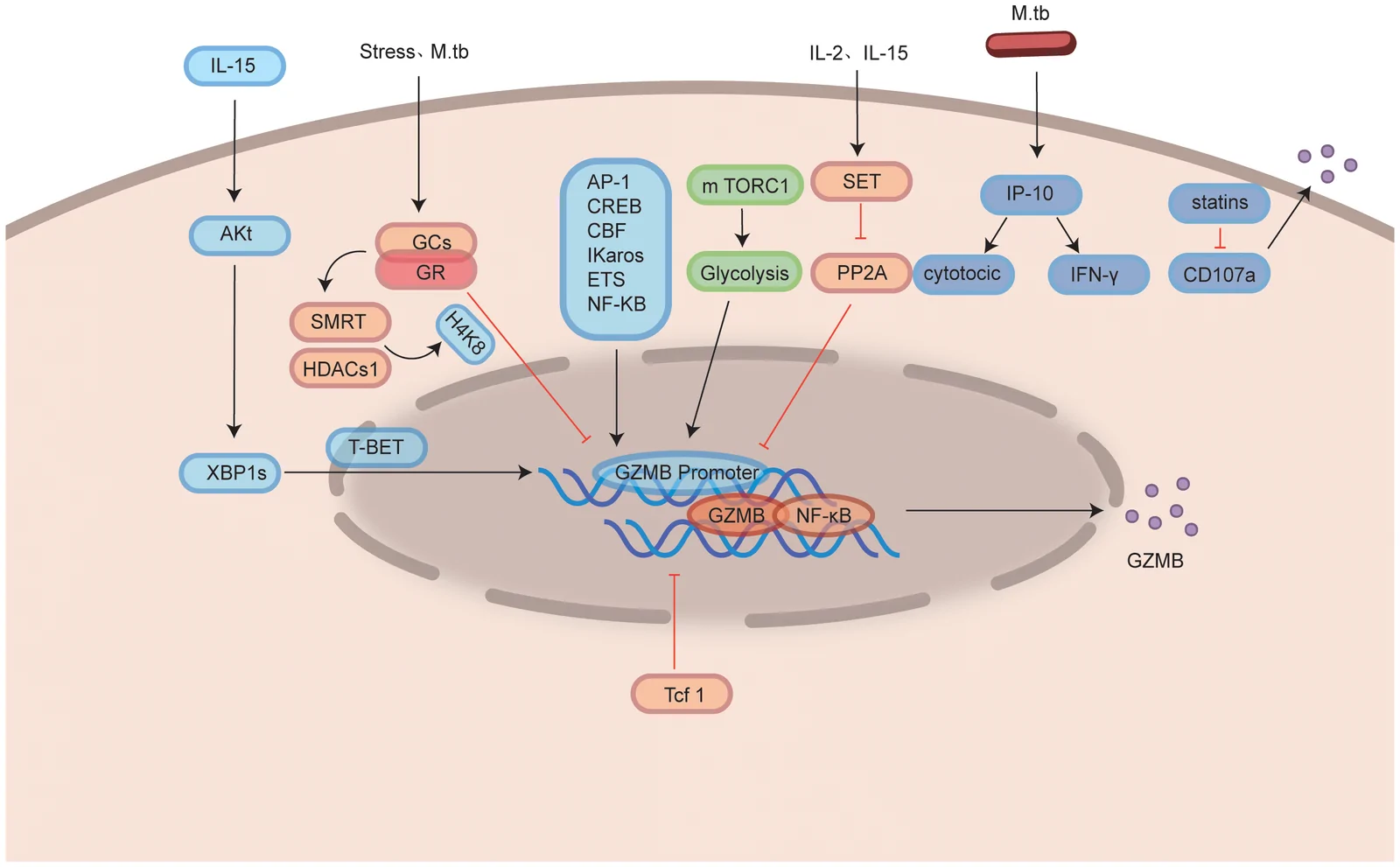
Expression and regulation of GZMB.

GZMB is mainly stored in the cytoplasmic granules of NK cells. After recognizing target cells, it is released into the immune synapse via exocytosis and synergistically induces apoptosis in target cells with perforin (PRF). Although the mechanism of GZMB-mediated target cell killing has been extensively studied, the regulatory mechanisms of its own expression and release have only recently received attention (**Figure 1**). Current research indicates that GZMB is finely regulated at multiple levels, including transcriptional regulation, epigenetic modification, metabolic reprogramming, and degranulation. Granzyme B (GZMB) is a key cytotoxic effector molecule responsible for NK cell-mediated antimicrobial and cytotoxic activity. GZMB expression is regulated at multiple levels, including transcriptional regulation, epigenetic modification, metabolic reprogramming, cytokine signaling, phosphatase-dependent pathways, and degranulation. IL-15 activates the Akt–XBP1s–T-BET signaling axis to promote GZMB transcription (36), while transcription factors including AP-1, CREB, CBF, Ikaros, ETS, and NF-κB further enhance GZMB promoter activity (37–39). Conversely, TCF1 negatively regulates GZMB transcription during NK-cell maturation (40). Epigenetically, glucocorticoid (GC)/glucocorticoid receptor (GR) signaling suppresses GZMB expression by recruiting HDAC1 and SMRT, reducing histone acetylation and chromatin accessibility (41). NK-cell metabolic reprogramming mediated by mTORC1-dependent glycolysis is required for optimal GZMB production, whereas inhibition of glycolysis impairs both GZMB and IFN-γ expression (42). The SET nuclear proto-oncogene (SET)–protein phosphatase 2A (PP2A) signaling axis positively regulates GZMB expression, while elevated IP-10(interferon gamma-induced protein 10) may suppress NK-cell cytotoxicity and IFN-γ production through CXCR3 signaling (43). Following transcription, GZMB is released through CD107a-associated degranulation, a process that can be inhibited by statins (44). Red arrows indicate inhibitory regulation, whereas black arrows represent activation or promotion.

## 3 NK cell recognition of *M.tb*

NK cells can be directly recognized and activated by *M. tb* and its cell wall components through a variety of surface receptors. Studies have shown that live BCG, heat-killed BCG, and *M.tb* can directly induce NK cell proliferation even in the absence of monocytes and exogenous cytokines such as IL-2, IL-12, IL-15, and IL-18 (45–47), and promote NK cells to express CD25 and CD69, secrete IFN-γ, and activate cytotoxicity. This process appears to depend on direct cell-to-cell contact (47).

NK cells have been reported to recognize mycobacterial components through Toll-like receptor 2 (TLR2) and NKp44. Based on these findings, it has been proposed that NK cells may initially recognize mycobacterial peptidoglycan (PG) or PG-associated components through TLR2, while IL-2 derived from peripheral T cells may contribute to NKp44 induction. Following the induction of NKp44 expression, NK cells may subsequently recognize mycobacterial arabinogalactan (AG) and mycolic acids (MA) through NKp44, potentially promoting further NK-cell activation. TLR2 participates in mycobacterial recognition by antigen-presenting cells and may also serve as an important receptor for the recognition of *M. tuberculosis*-derived components by NK cells. TLR2-Fc chimeric proteins bind to *M. tuberculosis* mAGP and PG, but not to AG or MA (48, 49). NKp44-Fc chimeras can bind to mycobacteria such as BCG and H37Rv, as well as mAGP, MA, and AG of *M.tb* (50).Of these four *M.tb* cellular components, only mAGP can independently activate NK cells to express CD25, CD69, and NKp44. When mAGP is co-cultured with resting NK cells, the addition of a TLR2 inhibitor can inhibit CD69 expression and prevent the production of IFN-γ, while the addition of an NKp44 inhibitor does not have such an inhibitory effect, indicating that NKp44 is not expressed or is poorly expressed on resting NK cells. NKp44 expression increases only after stimulation with BCG, mycolyl-arabinogalactan-peptidoglycan (mAGP), IL-2, or IL-15 (49–51). TLR2 is constitutively expressed on the surface of NK cells and mediates the recognition of mycobacterial components. After NKp44 expression is induced following activation, NK cells can recognize *M. tuberculosis* cell wall components through NKp44 (49). Therefore, in IL-2-preactivated NK cells, subsequent stimulation with M. tuberculosis-derived mAGP or live BCG in the presence of an NKp44-blocking antibody significantly reduced CD69 expression, suggesting that NKp44 contributes to NK-cell activation following secondary stimulation with mycobacterial components (49). The sequential recognition mechanism mediated by TLR2 and NKp44 suggests that NK-cell responses to mycobacterial components may involve a phased activation process. However, whether this sequential activation ultimately enhances, limits, or otherwise modulates host protective immunity against mycobacteria remains to be determined. Some studies have found that BCG stimulation can affect the function of NKp44+CD56bright NK cells and induce their apoptosis, thereby reducing the number of this subset (22). Since this study used the attenuated live vaccine strain BCG, whether these phenomena also occur during *M.tb* infection and their biological significance remain to be further investigated. In addition, ManLAM, as an important cell wall antigen of *M.tb* (52), can be recognized by macrophages, DC cells, neutrophils, natural killer T cells, T cells and B cells. Although there are no reports of it being recognized by NK cells, NK cells also express TLR2 and Mannose receptor(MR) receptors (53). Therefore, it is possible that NK cells can also recognize or be regulated by ManLAM of *M.tb*.

## 4 Inhibition and killing of *M.tb* by NK cells

NK cells release granulysin (GNLY), granzyme B (GZMB), and perforin (PFN), which contribute to the control of extracellular and intracellular M. tuberculosis. In addition, GZMB and PFN can induce apoptosis of M. tuberculosis-infected macrophages, a host-protective process associated with reduced intracellular bacterial viability and limitation of bacterial dissemination (54, 55). Cytokines such as IFN-γ and TNF-α secreted by NK cells can enhance the antibacterial ability of infected monocytes (30). In addition, NK cells can directly contact target cells through different receptors, inducing apoptosis or further enhancing the antibacterial ability of target cells.

### 4.1 NK cells secrete GNLY, GZMB, and PFN to kill *M.tb*

**Figure 2:**
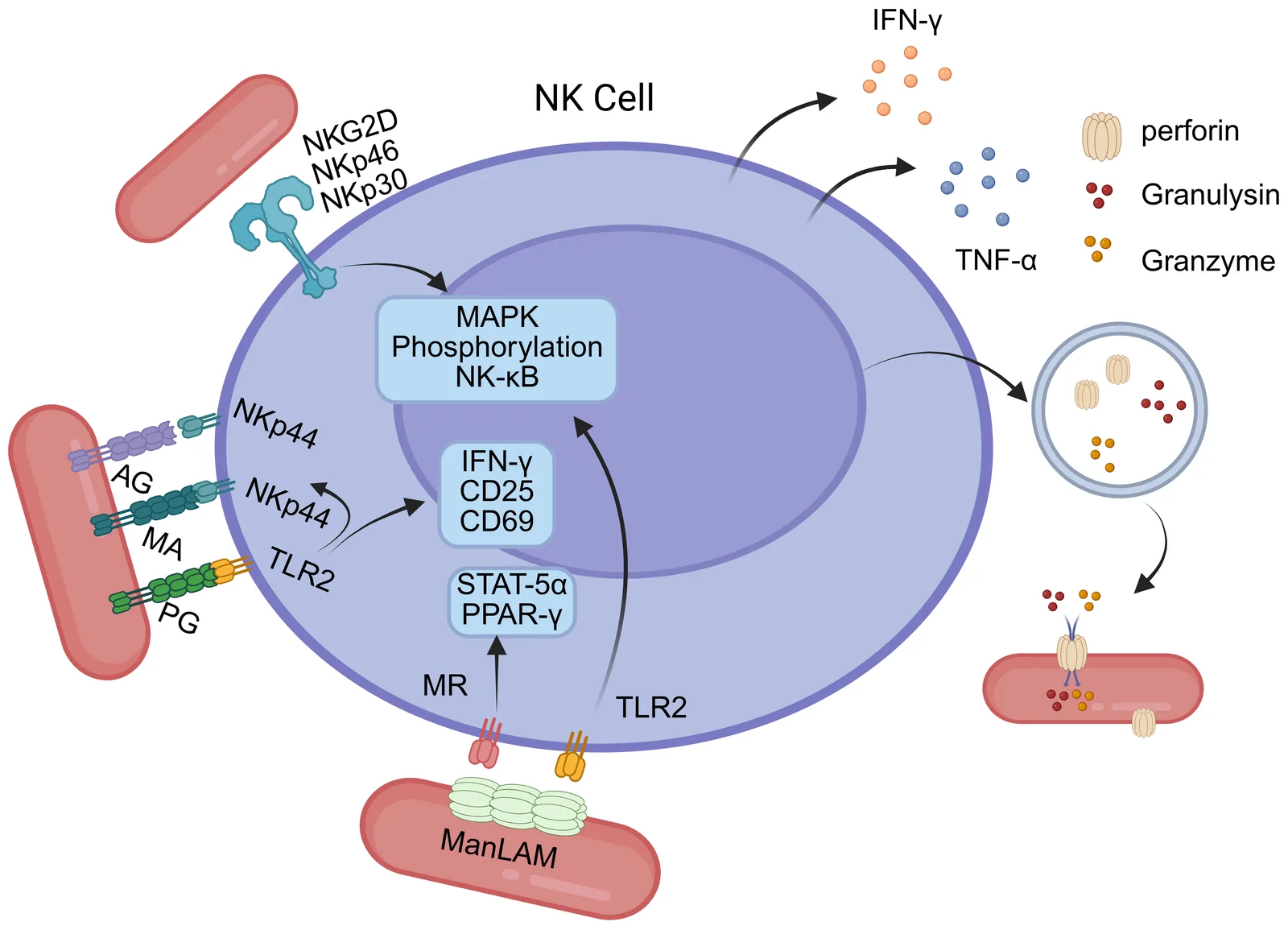
NK cells recognize and directly kill Mycobacterium tuberculosis. NK cells recognize Mycobacterium tuberculosis (M.tb) and its cell wall components through a variety of activation receptors, including NKp30, NKp44, NKp46, NKG2D, TLR2, and mannose receptor (MR), thereby activating intracellular signaling pathways such as the MAPK and NF-κB pathways. These signaling pathways promote NK cell activation, cytokine production, and cytotoxic responses. Activated NK cells secrete pro-inflammatory cytokines IFN-γ and TNF-α. Simultaneously, NK cells release cytotoxic granules containing perforin (PFN), granzyme (GNLY), and granzyme (GZM). PFN forms channels on the target cell membrane, promoting the intracellular delivery of GNLY and GZMs. GNLY directly disrupts the mycobacterial cell envelope, while GZM induces apoptosis in infected host cells through caspase-dependent and mitochondrial pathways, and may also directly target mycobacterial components. In summary, receptor-mediated activation, cytokine secretion, and granule exocytosis are the main mechanisms by which NK cells participate in the host’s defense against M.tb infection. Created in BioRender.

When inactivated NK cells are co-cultured with *M.tb* and can come into contact with each other, the number of *M.tb* in the culture medium is significantly reduced (56). Electron microscopy revealed that *M.tb* can bind through nanotube-like structures on the surface of NK cell walls, and PFN accumulates significantly at the contact sites between NK cells and *M.tb*. Furthermore, by activating the NKG2D and NCR receptors, PFN and GNLY are increased by activating the p38, ERK, and MAPK pathways (56). Electron microscopy showed that co-culturing *M.tb* with GNLY or PFN can cause damage to the *M.tb* cell wall (56, 57), while GZMB can attack multiple bacterial components of *M.tb* (58). GNLY can be secreted by activated NK cells and cytotoxic T lymphocytes, has cytotoxic and pro-inflammatory functions, and can kill by disrupting lipid metabolism and damaging the cell wall (59). PFN belongs to the membrane attack complex protein family and can insert into the target cell membrane to form a porous structure, mediating the entry of GNLY and GZMB into the target cell cytoplasm to achieve bactericidal function (60). In addition, PFN, GNLY, and GZMB are also key effector molecules of NK cell cytotoxicity. After entering target cells via PFN, GZMB can activate Caspase-3/7 and cleave BH3-interacting domain death agonist (Bid), leading to mitochondrial apoptosis and nuclear damage. In contrast, other granzymes may induce Caspase-independent cell death (61). Although GZMB can enter target cells independently of PFN, its function is affected (**Figure 2**).

### 4.2 NK cells secrete IFN-γ and TNF-α to enhance the antibacterial ability of macrophages

Interferon released by NK cells can trigger multiple intracellular effector mechanisms of macrophages, such as activating NADPH oxidases (NOX1, 2) and NO synthase (NOS2), leading to the formation of ROS and reactive nitrogen species (RNS) (62, 63). IFN-γ can also upregulate the monocyte IgG Fc receptor FcγRI to increase phagocytosis-based opsonin activity (63). In addition, IFN-γ and TNF-α produced by NK cells can upregulate the expression of the target cell adhesion factor ICAM-1 by activating the NF-κB pathway in target cells. ICAM-1 increases the adhesion of NK cells to target cell pairs, thereby increasing NK cell cytotoxicity (64). Schroder et al. provided a detailed explanation of the mechanism of action of IFN-γ, especially its effect on macrophages (63) as shown in **Figure 2**.

### 4.3 Receptor–ligand interactions mediate NK-cell responses against *M.tb*-infected target cells in contact

Multiple *in vitro* studies have demonstrated that NK cells can lyse monocytes infected with *M.tb* (65), Mycobacterium avium (66), or BCG (66) through direct cell–cell contact, thereby exerting antimycobacterial activity. IL-2 and IL-12 further enhance this effect (25). Co-culturing isolated NK cells with *M.tb*-infected monocytes can significantly reduce intracellular *M.tb*.Brill et al. found that direct contact between NK cells and macrophages infected with M. tb. is essential for inhibiting bacterial growth, while co-culture supernatant does not produce a similar effect (67). Currently reported receptor-ligand interactions between NK cells and M. tb.-infected macrophages include FasL/Fas, CD40L/CD40, NKG2D/ULBP1, and NKp46/Vimentin.

Fas is a transmembrane death receptor in the TNF receptor family that mediates apoptosis upon engagement with its ligand FasL, which is expressed on NK cells (68). Most cells in the body can express Fas, which, upon binding to FasL, forms a death-inducing signal complex (DISC), activating caspase-8 and initiating the extramitochondrial apoptosis pathway. The binding of NK cell FasL to macrophage Fas can promote macrophage apoptosis, thereby inhibiting intracellular *M.tb* proliferation (69). In addition, the Fas-FasL-mediated killing effect can be enhanced by IFN-γ. However, M. tuberculosis (*M.tb*) can interfere with the Fas/FasL axis (70) to promote the survival of infected macrophages and facilitate bacterial persistence. *M.tb*-infected macrophages exhibit reduced Fas expression and consequently decreased susceptibility to FasL-induced apoptosis, which may favor intracellular *M.tb* survival (69). In addition, *M.tb* infection can induce FasL expression in macrophages, which may promote apoptosis of Fas-expressing lymphocytes and contribute to local immune evasion (71, 72).

The CD40/CD40L pathway is also involved in the interaction between NK cells and macrophages (73). After macrophages are stimulated by CD40L, the expression of CD80, CD86, CD40, as well as NO production, is upregulated. Meanwhile, NF-kB, ERK1/2, p38 and Akt signaling pathways are activated, ultimately triggering the secretion of multiple cytokines (74, 75). Scott et al. demonstrated that direct co-culture of NK cells with macrophages enhanced the activation of both cell types, whereas culture supernatants from either cell type alone were insufficient to reproduce this effect, indicating a contact-dependent mechanism (74). After knocking out CD40 or CD40L and then co-culturing, the phagocytic effect of macrophages was weakened, but the activation of NK cells was not affected (74). Furthermore, glutathione (GSH) can promote the expression of FasL and CD40L on natural killer (NK) cells, thereby enhancing the ability of NK cells to control intracellular *M.tb* (76–78). Separately, glutathione depletion has been reported in human immunodeficiency virus (HIV) infection (79). Given the important role of GSH in NK-cell-mediated antimycobacterial responses, impaired glutathione homeostasis during HIV infection may contribute to defective host control of M. tuberculosis. The potential role of GSH dysregulation in HIV-associated tuberculosis warrants further investigation.

**Figure 3:**
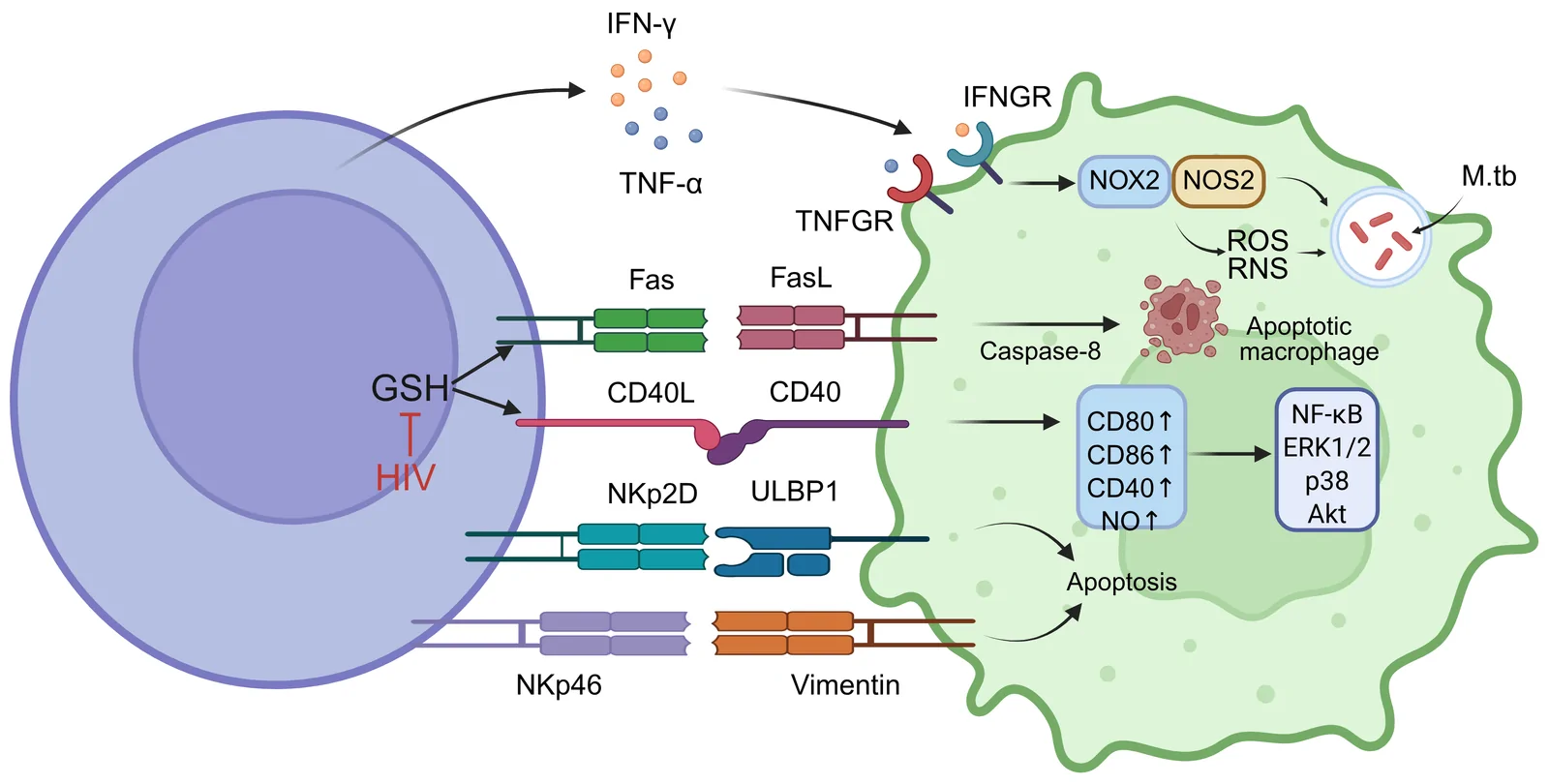
Interaction between NK cells and macrophages during Mycobacterium tuberculosis infection. NK cells recognize and interact with Mycobacterium tuberculosis (*M.tb*)-infected macrophages through multiple receptor–ligand pairs, including FasL/Fas, CD40L/CD40, NKG2D/ULBP1, and NKp46/Vimentin, leading to enhanced antimicrobial responses and cytotoxic activity. FasL expressed on NK cells binds to Fas on infected macrophages and induces caspase-8 activation and apoptosis, thereby restricting intracellular *M.tb* survival. CD40L–CD40 interaction promotes macrophage activation through NF-κB, ERK1/2, p38, and Akt signaling pathways, resulting in increased expression of costimulatory molecules (CD80, CD86, and CD40), nitric oxide (NO) production, and enhanced antimicrobial activity. NK-cell activating receptors NKG2D and NKp46 recognize stress-induced ligands on infected macrophages, including ULBP1 and vimentin, facilitating target-cell lysis. In addition, NK-cell-derived IFN-γ and TNF-α further potentiate macrophage antimicrobial functions by inducing NOX2/NOS2-dependent reactive oxygen and nitrogen species (ROS/RNS) production. HIV infection may impair these contact-dependent antimicrobial mechanisms by reducing glutathione (GSH)-dependent NK-cell activation and altering Fas/FasL signaling. Red lines indicate inhibitory regulation, whereas black arrows indicate activation or induction. Created in BioRender.

NKG2D and NKp46 are activating receptors on NK cells. Upon binding to ligands expressed on the macrophage surface, these receptors also mediate NK cell-mediated killing of M. tb-infected macrophages (20, 80). M. tuberculosis-infected monocytes upregulate ULBP1 and vimentin, ligands for the NK-cell activating receptors NKG2D and NKp46, respectively, thereby promoting NK-cell-mediated lysis. This cytotoxicity is enhanced by IL-2 or IL-12 and abrogated by blocking NKp46/NKG2D or their corresponding ligands (80, 81) (**Figure 3**).

Although NKp30 expression was also elevated, it did not affect NK-cell cytotoxicity against monocytes. Studies have shown that NKp30 mainly acts on immature dendritic cells (iDCs). NKp30 is involved in the lysis of uninfected iDCs mediated by NK cells. The binding of NKp30 to its ligand on iDCs can trigger NK cytotoxicity (82). In addition, NK cells from tuberculosis patients exposed to *M.tb*-infected monocytes showed lower NKp46 mRNA expression than those from healthy individuals, suggesting that tuberculosis may inhibit the NKp46 expression pathway in the body (10, 20).

## 5 NK cells inhibit *M.tb* growth by activating other immune cells

Compared to other pathogens, M. tb. appears to possess a unique ability to delay the adaptive immune response. NK cells can promote the migration of antigen-presenting dendritic cells (DCs) to lymphoid tissues in the early stages of infection, accelerating DC antigen presentation and T cell initiation. NK cells can also kill iDCs while preserving mDCs, thereby influencing antigen presentation and subsequent T-cell priming. Furthermore, NK cells finely regulate the adaptive immune response by upregulating CD8+ (83) and γδ+ T cell (84) activity and inhibiting the abnormal Treg expansion induced by M. tb (85).

### 5.1 DCs and NK cells

As the primary antigen-presenting cells, dendritic cells (DCs) mediate the cellular immune response to M. tb. Immature DCs (iDCs) are responsible for antigen uptake, and after maturation, they migrate to lymph nodes and activate T cells via MHC, co-stimulatory molecules, and cytokines (86). A bidirectional crosstalk exists between dendritic cells (DCs) and NK cells, whereby DCs can activate NK cells, while NK cells can reciprocally regulate DC maturation and function. This interaction contributes to the coordination of innate and adaptive immune responses and has been observed in both *in vitro* and *in vivo* settings (87–89). In the presence of M. tuberculosis as a maturation stimulus, iDCs enhanced NK-cell cytotoxicity, while activated NK cells reciprocally promoted DC maturation (88). Furthermore, NK cells activated by BCG-infected DCs can lyse uninfected iDCs, in part through NKp30, whereas DCs expressing high levels of HLA class I molecules are relatively resistant to NK-cell-mediated lysis (90). Thus, NK cells can selectively eliminate immature or otherwise susceptible DCs while sparing DCs that retain strong inhibitory signals, such as high levels of MHC class I molecules. This selective recognition is governed by the balance between activating and inhibitory receptor–ligand interactions and may contribute to NK-cell-mediated dendritic-cell editing and the subsequent regulation of antigen presentation during infection (91). However, these results are from *in vitro* experiments and have not yet been confirmed *in vivo*, although *in vivo* evidence of NK cell activation of DCs has been confirmed in viral infections (92).

Besides regulating antigen-mediated responses in dendritic cells (DCs), NK cells also produce IFN-γ, which is crucial for DC function. In the absence of IFN-γ, DCs primarily secrete IL-23 or IL-10, producing only a small amount of IL-12p70 (76). The addition of IFN-γ significantly increases IL-12p70 and promotes Th1 polarization (76). Further research by Keegan et al. showed that M. tb tRNA can be recognized by DCs and macrophages’ TLR8, producing IL-18 and IL-12p70, thereby stimulating NK cells to produce IFN-γ and forming a positive feedback loop that promotes Th1 responses (77).

During pulmonary M. tuberculosis infection, NK cells accumulate at the site of infection and become activated, where they can interact with DCs and contribute to DC maturation and subsequent T-cell priming (88, 93). In turn, T-cell-derived IL-2 can activate CD56bright NK cells, while M. tuberculosis-derived signals can induce an IL-12p70–IL-18–IFN-γ feedback loop in which NK-cell-derived IFN-γ further enhances IL-12p70 production, thereby promoting Th1-type immunity (77, 94).

### 5.2 T cells and NK cells

NK cells can regulate T cell function in tuberculosis infection. *In vitro* studies have shown that NK cells can promote the expansion of *M.tb*-stimulated γδ T cells and *M.tb*-specific CD8+ T cells (83, 84), and their effect is mainly dependent on IFN-γ, but can also be achieved through CD54-mediated cell contact.

NK cells also participate in the regulation of Tregs. Tregs are present in the blood and lesions of tuberculosis patients, and their number is negatively correlated with *M.tb*-specific immunity (83, 95). They can limit excessive inflammation, but may also weaken host immunity due to response imbalance (96, 97). NK cells can selectively cleave *M.tb*-induced Treg expansion through NKG2D and NKp46, thereby inhibiting excessive immune downregulation, but have no significant effect on non-*M.tb*-specific Tregs (85, 98).

## 6 NK-cell dysfunction in HIV-associated tuberculosis

HIV infection can further impair NK-cell-mediated immunity during tuberculosis. Compared with TB alone, HIV/TB co-infection is associated with alterations in NK-cell subset distribution, receptor expression, and effector functions. In particular, an increased proportion of CD56^neg NK cells has been observed in individuals with active TB and HIV co-infection, and this dysfunctional NK-cell subset may contribute to impaired antimycobacterial responses. Anti-TB treatment can partially restore NK-cell numbers and subset distribution, suggesting that these alterations are associated with disease activity (99).

HIV infection may also impair M. tuberculosis-responsive NK-cell functions. In individuals with HIV and latent TB infection, stimulation with M. tuberculosis antigens ESAT-6 and CFP-10 resulted in impaired expansion and functional responses of memory-like NK cells, accompanied by reduced IFN-γ, IL-15, and IL-32α responses and impaired NK-cell–monocyte interactions (100). In addition, HIV-associated glutathione depletion may further compromise NK-cell-mediated control of intracellular M. tuberculosis by reducing NK-cell effector functions (79). Collectively, these findings indicate that HIV/TB co-infection is associated with distinct NK-cell dysfunction that may compromise antimycobacterial immunity beyond that observed in TB alone.

## 7 Summary

NK cells play a crucial role in multiple key stages of *M.tb* infection. They not only directly participate in the recognition and clearance of *M.tb* as important innate effector cells, but also shape adaptive and innate immune responses through interactions with DCs, T cells, Tregs, and other immune cell populations. Numerous clinical studies have shown that with the progression of tuberculosis, NK cells exhibit significant exhaustion in terms of quantity, subset composition, and effector function. These characteristics include downregulation of activation receptors, decreased cytotoxicity, decreased IFN-γ secretion, and increased expression of inhibitory molecules. This suggests that NK-cell dysfunction may be an important immunological factor contributing to the persistence of *M.tb* infection. Currently, the mechanism by which *M.tb* induces NK cell exhaustion is not fully understood, but factors such as immune checkpoints, immunosuppressive cytokines, and metabolic reprogramming may all be involved. Further research integrating single-cell sequencing, spatial transcriptomics, and *in vivo* functional models is required to elucidate the dynamic changes of NK cells during different stages of infection and within distinct tissue microenvironments. Such studies may facilitate the development of host-directed therapies targeting NK-cell restoration or remodeling for drug-resistant and complicated tuberculosis.
